# Investigating #vapingcessation in Twitter

**DOI:** 10.21203/rs.3.rs-2976095/v1

**Published:** 2023-06-05

**Authors:** Samia Amin, Aditi Jaiswal, Peter Y Washington, Pallav Pokhrel

## Abstract

Evidence suggests that an increasing number of e-cigarette users report intentions and attempts to quit vaping. Since exposure to e-cigarette-related content on social media may influence e-cigarette and other tobacco product use, including potentially e-cigarette cessation, we aimed to explore vaping cessation-related posts on Twitter by utilizing a mixed-methods approach. We collected tweets pertaining to vaping cessation for the time period between January 2022 and December 2022 using snscrape. Tweets were scraped for the following hashtags: #vapingcessation, #quitvaping, and #stopJuuling. Data were analysed using Azure Machine Learning and Nvivo 12 software. Sentiment analysis revealed that vaping cessation-related tweets typically embody positive sentiment and are mostly produced in the U.S. and Australia. Our qualitative analysis identified six emerging themes: vaping cessation support, promotion of vaping cessation, barriers and benefits to vaping cessation, personal vaping cessation, and usefulness of peer support for vaping cessation. Our findings imply that improved dissemination of evidence-based vaping cessation strategies to a broad audience through Twitter may promote vaping cessation at the population level.

## What is already known on this topic

- Twitter-based study on e-cigarettes found that there are more positive than negative sentiments about e-cigarettes prevalent on the platform. However, there has been no research on the content related to vaping cessation on Twitter.
- We investigated sentiments and information dissemination concerning vaping cessation using Twitter data.

#### What this study adds

- Tweets with vaping cessation hashtags typically expressed positive sentiments and were mostly tweeted from the U.S. and Australia.
- The emergent themes in our study included vaping cessation support, promotion of vaping cessation, barriers and benefits to vaping cessation, personal vaping cessation, and usefulness of peer support for vaping cessation.

#### How this study might affect research, practice, or policy

- Twitter is an affordable tool with the potential to circulate evidence-based information on vaping cessation to the broader community on a global scale.

## BACKGROUND

E-cigarettes serve as an alternative to combustible tobacco products and individuals may vape (i.e., use e-cigarettes) to stop smoking cigarettes. However, conclusive evidence regarding vaping as an effective smoking cessation tool is lacking .[1] Individuals who use nicotine (known addictive substance in tobacco product) containing e-cigarettes regularly often become dependent on e-cigarette use and intend to quit.[2] Furthermore, studies show that e-cigarette vapor contains carcinogenic carbonyl compounds.[3,4] E-cigarette use may be linked with lung and bladder cancer and respiratory disorders.[5,6] Thus, concerns for health may also motivate regular, exclusive e-cigarette users to quit e-cigarette use.[7] For the past several years, e-cigarette content on social media has been widely prevalent and, hence, understanding how the topic of vaping cessation is represented on social media is vital.[8] Prior research has yet to address how individuals search and share vaping cessation concerns on social media.

Twitter is a microblogging and social networking platform. Unstructured, free-text tweets relating to health care are frequently shared on Twitter.[9] Sentiment analysis is a natural language processing technique that enables the analysis text for the intensity of sentiment, which in turn facilitates the characterization of discussions pertaining to various health-related issues.[10,11] Sentiment analysis uses computational algorithms to extract subjective information from written text and to identify the strength of the text's positive or negative tone.[12-14] Sentiment analysis has been used to predict health behaviours through an increased understanding of how people feel with respect to specific health topics or conditions.[15-17]

A recent Twitter-based study on e-cigarettes found that there are more positive than negative sentiments about e-cigarettes prevalent on the platform.[18] However, there has been no research on the content related to vaping cessation on Twitter. Examining how vaping cessation is represented on Twitter is imperative because social media platforms such as Twitter may influence an individual's decision to continue using e-cigarettes.[19-22] Twitter content may not only provide factual information about vaping (e.g., adverse consequences) but also express sentiments through tone or underlying message connotations. Gaining a deeper understanding of the sentiments prevalent on Twitter regarding vaping cessation may assist public health professionals and policymakers in better utilizing social media platforms to disseminate evidence-based vaping cessation strategies. For example, a finding that sentiments tied to vaping cessation are generally positive may suggest higher tendency among users on the platform to engage in discussions about vaping cessation and also higher receptivity towards vaping cessation messages.  Thus, the objective of this study was to apply a mixed-methods approach to analyze the sentiments expressed in Twitter posts concerning vaping cessation. Specifically, we examined Twitter posts under the following hashtags: #vapingcessation OR #quitvaping OR #stopJuuling.

## METHODS

The process of extracting data from a website is commonly referred to as web scraping. In this study, tweets were manually collected using snscrape,[23] a Python based library that allows for the extraction of tweets without the need for personal Twitter API keys. The library provides a powerful search functionality to help filter tweets based on various conditions, such as date-time, language, and number of likes. For this study, we obtained English tweets related to vaping cessation by using the search query #vapingcessation OR #quitvaping OR #stopJuuling and setting the "since" and "until" flags to January 1, 2022 and December 31, 2022, respectively. Through this we obtained 405 publicly accessible vaping cessation-related full-text tweets with their metadata such as the number of followers and user geolocation. Identifiable information was not accessible and was therefore not examined.

Data were downloaded in the form of CSV (comma separated value) file. The retrieved data were cleaned by removing the duplicate tweets, tweets containing URL(s) or hashtags only, and non-English tweets. The cleaning process resulted in an analytical sample of 231 tweets. We focused on the analysis of the textual information in the tweets and did not analyse photos and videos, as visual information is not integral to the communication in Twitter.

This study was conducted in two parts. First, to understand sentiments expressed towards vaping cessation, sentiment analysis was performed using Azure Machine Learning,[24] a popular open source tool designed to estimate the strength of positive and negative sentiment in short, informal text. It emits the text sentiment as a numerical rating known as a 'sentiment score' ranging between 0 and 1. A score closer to 0 it is indicative of a negative sentiment while a score closer to 1 is indicative of a positive sentiment. Positive numbers indicate favourable attitudes, while negative numbers indicate negative attitudes.[24] Intermediate values are tagged as neutral.

Thematic content analysis, performed using Nvivo 12,[25] was used to generate codes through an inductive approach to identify different themes represented in the data. Initially, the raw data (tweets) were reviewed to gain familiarity, and later, the tweets were inductively coded according to their meanings within the corresponding themes. For each theme, tweets were organized within categories according to emerging patterns. Themes and categories were not mutually exclusive. A tweet may be coded into one or more themes or categories. The themes' titles were adjusted according to the findings.

## RESULTS

According to our automated sentiment analysis pipeline, a total of 69% (160 of 231) tweets represented positive sentiment, 16% negative (n = 37), and 15% neutral (n = 34). Across accounts, the number of followers ranged from 0 to 21,338. The mean number of followers was 1,332 ± 2904. 86% (189 of 231) of tweets were posted from the U.S. Other locations represented in our curated dataset included Australia, Canada, Japan, Malaysia, the United Arab Emirates, and the United Kingdom. The distributions of the positive, negative, and neutral sentiments are shown in Fig. 1. Most of the positive and neutral sentiment tweets originated from the U.S. and Australia, respectively. The negative sentiment tweets' locations varied across the globe.

The qualitative findings were grouped into 6 main emerging themes and several subthemes (Table 1) based on positive, negative, and neutral sentiments. The majority of the tweets corresponded to identified themes of vaping cessation support offers (43%) and promotion of vaping cessation (31%) (Fig. 2). The primary themes and subthemes are described below, and illustrative tweets related to each theme and subtheme are listed in Table 1.

Table 1
Themes and subthemes with relevant tweets

| Themes | Subthemes | Relevant Tweets |
|---|---|---|
| Quit vaping support | Quit coaching | Tweet 1: People who get support with quitting tobacco are more likely to succeed than those who try to quit alone. Get free, personalized support from our Quit Coaches to quit for good.<br><br>Tweet 2: ……has helped Californians quit for 30 years through free, customized one-on-one coaching that is grounded in science. Start your quit journey today<br><br>Tweet 3: You can do it! This Womens Health Fitness Day break free from tobacco! connect with a quit coach and take the first step toward improving your health and wellbeing<br><br>Tweet 4: This pride month and every month we are proud to be here with free quit help for MN LGBTQ + community. Reach a free quit coach |
| | Medications references | Tweet 5: Holiday stress? Nicotine can make it worse. Gift yourself free help to quit smoking or vaping by visiting for judgment-free coaching and patches, gum or lozenges.<br><br>Tweet 6: Fall fun is here, enjoy the season to the fullest with free quit coaching, nicotine patches and gum. Visit……. to get started. |
| | Rewards | Tweet 7: Ready to quit smoking or vaping? Our free quit program begins…... Free coaching, nicotine patches, and rewards for program completion including a Fit Bit Watch! |
| | Use of digital technologies for quitting | Tweet 8: Have you heard of the free mobile app @…....? It helps users Quit vaping by tracking their consumption of vaping Products and recognizing triggers that may give them the urge to vape. Click the link to download the app or for more info!<br><br>Tweet 9: Want help quitting vaping? @...... is a free, text-to-quit program for young people ages 13–24. Join the hundreds of thousands of young people getting help from This is Quitting!<br><br>Tweet 10: Great News! A researcher at …... starting a project to help teens stop vaping! This project will use an adapted version of the smart phone app @ ……... in order to help younger people quit vaping. |
| | Navigating the website for quit service | Tweet 11: 70% of MN students who vape show signs of dependence. Want free help? Text to 36072 to chat with a coach and get confidential help from…………….. Minnesota's quit program just for teens.<br><br>Tweet 12: Have you heard of New Mexico's latest cessation program…….? It is a national text-based program aimed to help teens quit vaping<br><br>Tweet 13: Tobacco Free Florida in Okaloosa has introduced ………… a first-ever text-based program offering tools and resources to address teen e-cigarette use. |

| Themes | Subthemes | Relevant Tweets |
|---|---|---|
| | Quit line assistance | Tweet 14: Did you know that your local 211 can connect you to quit programs if you are looking to quit smoking, vaping, or stop using tobacco products? Call ……...or text your zip code to ………to learn more.<br><br>Tweet 15: A nicotine-free vape is not a worry-free vape. According to the FDA, vapes can expose you to toxic chemicals like formaldehyde and acrolein, even if they don't have nicotine. The Maryland Tobacco Quitline is a free service……. Call……….. |
| Promotion of vaping cessation | Year event | Tweet 16: 4/16 is National Stress Awareness Day. While stress develops from multiple avenues, research shows youth who vape are at higher risk for irritability, anxiety, and mood swings. Visit ……<br><br>Tweet 17: For International Youth Day, we want to highlight ……... Their mission is to change the tobacco use culture in California high schools, colleges, & other youth communities. Check out their digital media graphics collection. |
| | Health issues | Tweet 18: Concerned about your lung health? Your overall health? Ready to quit vaping……. Call …...or visit…… for free help<br><br>Tweet 19: Need a reason to quit vaping? researchers report that the use of e-cigarettes that contain nicotine can increase blood clotting; deterioration in small blood vessels! these effects are similar to those caused by traditional cigarettes. thoughts? |
| | Scientific evidence | Tweet 20: Do you think long term vapers are interested in quitting? Findings from an online survey conducted in 2017 discovered that only 25–50% of participants had initiated vaping cessation! do you think more vape users are interested in quitting today? |
| | Teen quit | Tweet 21: Want to help teens quit e-cigarettes? Order FDA free new toolkit on youth vaping here……... and get the facts………….<br><br>Tweet 22: Did you know? 34% of New Mexico high school youth reported currently using tobacco products (including e-cigarettes). ……………program provides resources and support to quit vaping for good. Learn more…………. |
| | School | Tweet 23: Did you know that more than 2 million middle and high school students use e-cigarettes in 2021? The Health Department of Northwest Michigan is providing……….. & vape disposal boxes at schools as a way to encourage students to quit vaping.<br><br>Tweet 24: Canberra schools have begun installing vape detectors to curb the harmful habit among students. What do you think of this initiative? |
| | Video | Tweet 25: What happens when a teen starts vaping? In this video from……... pediatricians explain the health effects of e-cigarette use. Know the risks! |

| Themes | Subthemes | Relevant Tweets |
|---|---|---|
| | Environmental issue | Tweet 26: Vaping is bad for your health; bad for the environment. Vaping has led to an increase in plastic waste, tech waste; hazardous waste…………….. |
| | | Tweet 27: Happy Earth Day! Help reduce the environmental impacts of smoking & vaping by quitting today! Visit ……. to learn how you can quit |
| | | Tweet 28: This year, the equivalent of 1,200 car batteries worth of lithium is on track to end up in UK landfill in the form of disposable e-cigarette. |
| Quit barrier | Lack of motivation | Tweet 29: You're not alone. Many others are taking the first step to quitting vaping. Free help for youth and young adults ages 13–24 is available by…… |
| | | Tweet 30: I forgot my puff at home and time has never moved this slow at my job. I feel really good actually but Idk I just know it would a been 11pm like 3 hours ago if I had it. |
| | Nicotine craving | Tweet 31: I did not realize how hard trying to quit vaping is, the cravings are horrible |
| | | Tweet 32: It’s been 2 weeks since …. stopped vaping………… I’m having a really bad craving……. |
| | Withdrawal symptoms | Tweet 33: Day 1 of not vaping, just took my last puff, starting now, I am officially done with it, it hurts to breathe or do anything. |
| | Anxiety | Tweet 34: While vaping may feel like it helps you alleviate stress and anxiety, it actually makes feelings of anxiety and depression worse. Use our free resources to quit vaping………… |
| Personal quit experience | | Tweet 35: Today I quit vaping. Smoking has been part of my life for the past 18 years. I want to make this public commitment to myself stop smoking for good! |
| | | Tweet 36: 2 weeks vape free. Feeling better than ever. |
| | | Tweet 37: So, after 20yrs of smoking cigarettes I quit for vaping and after 4 years of vaping I quit for life today. day1 quit vaping |
| Peer support | | Tweet 38: 1-week vaping/nicotine free. I had not gone a single day without nicotine since I was 17, before this. I am really proud of myself and my quitting partner whose helped me a lot. I never thought I'd make it past a couple hours. But, I actually quit vaping.06/…/2022- quit date |
| | | Tweet 39: Are you a parent of a teen who might be vaping and would like them to quit? There is a free online tool called the ………. created by …………. researchers! Click the link to learn about this resource! |
| | | Tweet 40: Every quit journey starts with a first step. Set a quit date. Next, if you have trusted friends, family, and co-workers, let them know. They can support you to quit |

| Themes | Subthemes | Relevant Tweets |
|---|---|---|
| Benefits of quitting | | Tweet 41: Quitting tobacco or vaping products can be a big step to better health, but just as important is planning how to quit…… experts offer these helpful tips to help you prepare to quit successfully.<br><br>Tweet 42: Vaping is associated with stunted brain development, heart damage, withdrawal symptoms, and other adverse health effects. So, what do you do when it's time to quit vaping? You can start here…... |

#### Theme 1: Vaping cessation support offer

Several motifs related to vaping cessation support were revealed from the positive sentiment tweets such as quit coaching, medication references, rewards for cessation, use of digital technologies for quitting, navigating the website for quit services, and assistance with quit lines. For example, free quit vaping coaching was offered by directing individuals seeking quit assistance to the specific state-based support (e.g., California or Minnesota) or by advising them to develop personalized (e.g., women or LGBTQ) quit plans with the help of an expert quit coach. Several tweets mentioned offering free quit aids (nicotine patches, gum, or lozenges) or rewards (e.g., Fit Bit watch). Digital technology such as puff counting (e.g., Puff count app) or text message-based support systems by a mobile app (e.g., This is Quitting or Quit2Heal) for vaping cessation support was mentioned in several tweets. Few tweets included links to nationwide (e.g., My Life My Quit) or state-based (e.g., New Mexico Department of Health or Florida) websites (e.g., Live Vape Free) for vaping cessation services. Information about several free quit assistance support services (e.g., Indiana Quitline, Maryland tobacco Quitline) were also offered.

#### Theme 2: Promotion of vaping cessation

The promotion of vaping cessation was emphasized in several tweets celebrating different types of annual events or occasions such as the International Youth Day, National Sons and Daughter Day, Best Friend Day, Happy Earth Day, National Stress Awareness Day, American Health Month, Women's Fitness Day, National Compliment day, and World No Tobacco Day. To encourage individuals to quit vaping, a number of health-related issues were emphasized, including lung health, mental health (depression, stress & anxiety), substance use disorder, blood clotting, pregnancy status, sleep, nystagmus, and eye cancer. Few tweets endorsed vaping cessation by posting scientific literature. The United States Food and Drug Administration (FDA) approved free quit vaping tool kits, various quit intervention programs, and literature on addiction, nicotine, and brain development-related tweets also promoted teen vaping cessation. The role of the school in promoting vaping cessation was tweeted in terms of establishing vape disposal boxes or installation of vape detectors. A few evidences based videos were also posted to persuade individuals to quit vaping. Environment-related tweets that mentioned vaping devices as a detrimental source of lithium waste in landfills, and climate change due to exhaled vapor from e-cigarettes, also encouraged quitting vaping.

#### Theme 3: Barriers to vaping cessation

The tweets with negative sentiments highlighted fear of anxiety, nicotine craving, withdrawal symptoms, and lack of motivation as barriers to quitting vaping. The primary reasons for the lack of motivation to stop vaping were lack of access to a personal vaping device or attempts at quitting without any professional support or assistance (i.e., quitting cold turkey). Numerous tweets mentioned intense nicotine craving as the cause of the failure to successfully stop vaping. Physical symptoms that were identified in the tweets as a barrier to quitting vaping were sore throats, short breaths, and lung scarring. Inability to manage anxiety was also another barrier to quitting vaping.

#### Theme 4: Personal vaping cessation experience

A total of 7.7% (18 of 231) tweets shared personal timelines of remaining vape free, which ranged from 6 hours to 150 days. Some tweets mentioned the personal excitement and struggles of the vape-free journey. Some tweets shared individuals' information-seeking quit strategies.

#### Theme 5: Peer support for vaping cessation

The importance of peers and family members (e.g., spouse, partner, friends, parents, grandparents, co-workers) in vaping cessation was another prominent theme. Tweets suggested that sharing a quit date with others in one's social network might be a great way to get support from them. Teens might get direct support from their parents, grandparents or school. Young adults might get support from their partners who intend to help them to quit vaping.

#### Theme 6: Benefits to vaping cessation

Tweet contents revealed various benefits of quitting vaping such as reduced stroke risk, proper teen brain development, lower blood pressure, mode improvements, and improved heart rates and heart health. Few tweets offered helpful tips for a successful quit. The impact of second and third-hand vaping was also stated in a few tweets.

### DISCUSSION

This may be one of the first studies to analyze the sentiments expressed on Twitter posts about quitting e-cigarette use or vaping cessation. The study focused specifically on the following hashtags: #vapingcessation, #quitvaping, and #stopJuuling. The sentiment analysis revealed that tweets with vaping cessation hashtags typically expressed positive sentiments and were mostly tweeted from the U.S. and Australia. The prevalence of negative and neutral sentiments proportion was almost similar (16% and 14% respectively) and substantially lower than positive sentiments. The qualitative analysis identified 6 emergent themes: vaping cessation support offers, promotion of vaping cessation, barriers to vaping cessation, benefits of vaping cessation, personal vaping cessation experience, and peer support for vaping cessation. Our findings suggest that Twitter is a useful tool to identify vaping cessation-related sentiments and themes commonly expressed in the public.

Our findings suggest that the majority of the posts related to vaping cessation hashtags were tweeted in the United States and Australia, which indicates a higher interest in vaping cessation in these geographical locations. Regulations on nicotine-containing e-cigarettes differ globally.[26] Across all Australian States and Territories, it is illegal to sell nicotine-containing e-cigarettes because liquid nicotine is classified as a 'Schedule 7-Dangerous Poison'; however, users can legally import nicotine-containing e-cigarettes through the Personal Importation Scheme which states that users must obtain a prescription from a physician.[27] Australia has relatively low rates of e-cigarette use compared to other countries,[28] which is likely a result of this regulatory policy. However, anyone 21 years of age or older in the U.S. can purchase nicotine-containing e-cigarettes,[29] which are highly addictive.[30] Given the higher prevalence of e-cigarette use in the U.S. and relatively freer access to nicotine-containing e-cigarette products, a multipronged approach to counter e-cigarette use through prevention and cessation may be particularly useful in the U.S. Such an approach may benefit from strategically utilizing social media platforms such as Twitter.

Our current study presents a timely analysis of Twitter handles. Thus, Twitter might be a particularly promising digital platform for delivering vaping cessation interventions to a broader community as prior Twitter-delivered interventions for smoking demonstrate promise.[31] Additionally, Twitter can allow private groups to be created, making it ideal for delivering and privatizing a vaping cessation intervention to those who will seek such quitting support. Evidence also suggests that Twitter-based social media interventions that combine traditional online social support with daily auto-messages is novel for tobacco product use cessation.[31] Hence, Twitter is an affordable tool with the potential to circulate evidence-based information on vaping cessation to the broader community on a global scale. Future studies are encouraged to explore how to integrate other social media platforms such as Instagram or TikTok with vaping cessation interventions.

The emergent themes related to vaping cessation hashtags on Twitter as highlighted by the present data are consistent with previous qualitative Twitter-based studies which have described similar themes related to barriers, promotion, and motivation to quit smoking or e-cigarette use.[32–36] However, the current qualitative study based on sentiment analysis may be the first study to focus on the unique themes associated with vaping cessation. The current qualitative findings may provide a "baseline" for future social media-based vaping cessation intervention programs.

There are several limitations to this study, including the use of hashtags for data collection. For example, many traditional smoking-related posts (such as #quitsmoking or #smokingcessation) might also include vaping cessation hashtags. Data pertaining to such hashtags might have been excluded from the current dataset, reducing the potentially eligible pool of tweets included in the current data analysis. Additionally, we might have missed relevant messages by limiting our search strategy to #vapingcessation, #quitvaping, and #stopJuuling. Many accounts did not publicly declare the locations of their origin, which precluded them from being included in our analysis. Further, we did not consider retweets of the eligible posts. Future studies should provide a better understanding of retweeting behaviour because retweeting is one of the primary modes of information dissemination on Twitter. Finally, we did not include media (pictures, videos, or emoji) related to #vapingcessation OR #quitvaping OR #stopJuuling on Twitter. Future studies should also consider analysing such media posts as prior studies have reported that video or emoji containing posts express positive sentiments and engage the users most in the social media.[37]

Despite the above-mentioned limitations, our findings demonstrate that vaping cessation hashtags on Twitter may have a lot of potential to promote cessation at the population level. Future research and policy efforts need to focus on increasing vapers' motivation to quit vaping using social media platforms. Additionally, those who are attempting to stop vaping may benefit from tailored Twitter-based treatment interventions. Moreover, to be effective, Twitter-based interventions may need to utilize peer or other social networks dynamics and address the physical and psychological consequences of vaping withdrawal.

## Declarations

**Ethics approval and consent to participate:** Not applicable

**Consent for publication:** Not applicable (No Human participants)

**Availability of data and materials:** The datasets used and/or analysed during the current study available from the corresponding author on reasonable request.

**Competing interests:** None

**Funding:** This research was supported by funds from the National Cancer Institute (R01CA228905)

**Authors' contributions:** SA and PP conceptualized the study. SA performed data analysis and prepared the first draft of the manuscript. AJ and PYW led data collection and provided conceptual and writing feedback. PP supervised the project and provided writing feedback.

**Acknowledgement:** Not applicable

## References

1. Wang, R. J., Bhadriraju, S., & Glantz, S. A. (2021). E-cigarette use and adult cigarette smoking cessation: a meta-analysis. *American Journal of Public Health*, 111(2), 230–246.
2. Palmer Palmer, A. M., Smith, T. T., Nahhas, G. J., Rojewski, A. M., Sanford, B. T., Carpenter, M. J., & Toll, B. A. (2021). Interest in quitting e-cigarettes among adult e-cigarette users with and without cigarette smoking history. *JAMA Network Open*, 4(4), e214146.

3. Kosmider, L., Kimber, C. F., Kurek, J., Corcoran, O., & Dawkins, L. E. (2018). Compensatory puffing with lower nicotine concentration e-liquids increases carbonyl exposure in e-cigarette aerosols. *Nicotine & Tobacco Research*, 20(8), 998–1003.
4. Sala, C., Medana, C., Pellegrino, R., Aigotti, R., Bello, F. D., Bianchi, G., & Davoli, E. (2017). Dynamic measurement of newly formed carbonyl compounds in vapors from electronic cigarettes. *European Journal of Mass Spectrometry*, 23(2), 64–69.
5. Herriges, M.J., Pinkhasov, R., Shapiro, O., Jacob, J.M., Basnet, A., Bratslavsky, G. & Goldberg, H., (2022). E-cigarette use and the risk of bladder and lung cancer. *Journal of Clinical Oncology*. 40:6_suppl, 443-443
6. Xie, W., Kathuria, H., Galiatsatos, P., Blaha, M. J., Hamburg, N. M., Robertson, R. M., Bhatnagar, A., Benjamin, E. J., & Stokes, A. C. (2020). Association of electronic cigarette use with incident respiratory conditions among US adults from 2013 to 2018. *JAMA Network Open*, 3(11), e2020816.
7. Kong, G., Bold, K. W., Cavallo, D. A., Davis, D. R., Jackson, A., & Krishnan-Sarin, S. (2021). Informing the development of adolescent e-cigarette cessation interventions: A qualitative study. *Addictive Behaviors*, 114, 106720.
8. McCausland, K., Maycock, B., Leaver, T., & Jancey, J. (2019). The messages presented in electronic cigarette-related social media promotions and discussion: scoping review. *Journal of Medical Internet Research*, 21(2), e11953.
9. Finfgeld-Connett D. (2015). Twitter and health science research. *Western Journal of Nursing Research*, 37(10), 1269–1283.
10. Salathé, M., & Khandelwal, S. (2011). Assessing vaccination sentiments with online social media: implications for infectious disease dynamics and control. *PLoS Computational Biology*, 7(10), e1002199.
11. Cole-Lewis, H., Varghese, A., Sanders, A., Schwarz, M., Pugatch, J., & Augustson, E. (2015). Assessing electronic cigarette-related tweets for sentiment and content using supervised machine learning. *Journal of Medical Internet Research*, 17(8), e208.
12. Beaunoyer, E., Arsenault, M., Lomanowska, A.M., Guitton, M.J., (2017) Understanding online health information: evaluation, tools, and strategies. *Patient Education and Counseling*, 100(2):183-189.
13. Gohil, S., Vuik, S., Darzi, A., (2018) Sentiment analysis of health care tweets: review of the methods used. *JMIR Public Health and Surveillance*, 4(2): e43.
14. Zorman, M., Verlic, M., (2009) Explanatory approach for evaluation of machine learning-induced knowledge. *Journal of International Medical Research*. 37(5):1543-1551.
15. D'Alfonso, S., Santesteban-Echarri, O., Rice, S., Wadley, G., Lederman, R., Miles, C., Gleeson, J., & Alvarez-Jimenez, M. (2017). Artificial intelligence-assisted online social therapy for youth mental health. *Frontiers in Psychology*, 8, 796.
16. Greaves, F., Ramirez-Cano, D., Millett, C., Darzi, A., & Donaldson, L. (2013). Use of sentiment analysis for capturing patient experience from free-text comments posted online. *Journal of Medical Internet Research*, 15(11), e239.

17. Mazzocut, M., Truccolo, I., Antonini, M., Rinaldi, F., Omero, P., Ferrarin, E., De Paoli, P., & Tasso, C. (2016). Web conversations about complementary and alternative medicines and cancer: content and sentiment analysis. *Journal of Medical Internet Research*, 18(6), e120.
18. Godea, A.K., Caragea, C., Bulgarov, F.A., Ramisetty-Mikler, S. (2015). An analysis of Twitter data on e-cigarette sentiments and promotion. In: Holmes, J., Bellazzi, R., Sacchi, L., Peek, N. (eds) *Artificial Intelligence in Medicine*. AIME 2015. Lecture Notes in Computer Science, vol 9105. Springer
19. Chmiel, A., Sienkiewicz, J., Thelwall, M., Paltoglou, G., Buckley, K., Kappas, A., & Hołyst, J. A. (2011). Collective emotions online and their influence on community life. *PloS One*, 6(7), e22207.
20. Oyeyemi, S. O., Gabarron, E., & Wynn, R. (2014). Ebola, Twitter, and misinformation: a dangerous combination? *British Medical Journal*. 349, g6178.
21. Wynn, R., Oyeyemi, S.O., Johnsen, J.A., & Gabarron, E., (2017) Tweets are not always supportive of patients with mental disorders. *International Journal of Integrated Care.*17(3): A149.
22. Dunn, A. G., Surian, D., Dalmazzo, J., Rezazadegan, D., Steffens, M., Dyda, A., Leask, J., Coiera, E., Dey, A., & Mandl, K. D. (2020). Limited role of bots in spreading vaccine-critical information among active Twitter Users in the United States: 2017-2019. *American Journal of Public Health*, 110(S3), S319–S325.
23. Snscrape. Available at: https://github.com/JustAnotherArchivist/snscrape
24. Harfoushi, O., Hasan, D., & Obiedat, R. (2018). Sentiment analysis algorithms through azure machine learning: analysis and comparison. *Modern Applied Science*; 12 (7): 49-58
25. QSR International. *QSR Nvivo* 12. Available at: http://www.qsrinternational.com/
26. Kennedy, R. D., Awopegba, A., De León, E., & Cohen, J. E. (2017). Global approaches to regulating electronic cigarettes. *Tobacco Control*, 26(4), 440–445.
27. Therapeutic Goods Administration. (2019). *Electronic cigarettes*. https://www.tga.gov.au/community-qa/electronic-cigarettes
28. Tehrani, H., Rajabi, A., Ghelichi- Ghojogh, M. et al. The prevalence of electronic cigarettes vaping globally: a systematic review and meta-analysis. *Archives of Public Health*. 80, 240 (2022).
29. Friedman, A. S. (2020). Tobacco-21 Laws: Insights from the US experience. *Nicotine & Tobacco Research*, 22(7), 1254–1255.
30. Benowitz, N. L. (2008). Clinical pharmacology of nicotine: implications for understanding, preventing, and treating tobacco addiction. *Clinical Pharmacology and Therapeutics*, 83(4), 531–541.
31. Pechmann, C., Pan, L., Delucchi, K., Lakon, C. M., & Prochaska, J. J. (2015). Development of a Twitter-based intervention for smoking cessation that encourages high-quality social media interactions via automessages. *Journal of Medical Internet Research*, 17(2), e50.
32. Krittanawong, C., & Wang, Z. (2017). Mining twitter to understand the smoking cessation barriers. *World Journal of Cardiology*, 9(10), 794–795.
33. Chean, K. Y., Goh, L. G., Liew, K. W., Tan, C. C., Choi, X. L., Tan, K. C., & Ooi, S. T. (2019). Barriers to smoking cessation: a qualitative study from the perspective of primary care in Malaysia. *British*

*medical journal open*, 9(7), e025491.

34. Hoek, J., Gifford, H., Maubach, N., & Newcombe, R. (2014). A qualitative analysis of messages to promote smoking cessation among pregnant women. *British medical journal open*, 4(11), e006716.
35. Cordon, M., Eyestone, E., Hutchison, S., Dunlap, D., Smith, L., Williams, R. M., Kim, E., Kao, J. Y., Hurtado-de-Mendoza, A., Stanton, C., Davis, K., Frey, J., McKee, B., Parikh, V., Taylor, K. L., & Lung Screening, Tobacco, and Health Study (2021). A qualitative study exploring older smokers' attitudes and motivation toward quitting during the COVID-19 pandemic. *Preventive Medicine Reports*, 22, 101359.
36. Hanafin, J., & Clancy, L. (2020). A qualitative study of e-cigarette use among young people in Ireland: Incentives, disincentives, and putative cessation. *PloS One*, 15(12), e0244203.
37. Bhattacharya, S., Srinivasan, P., & Polgreen, P. (2017). Social media engagement analysis of U.S. Federal health agencies on Facebook. *BMC Medical Informatics and Decision Making*, 17(1), 49.

## Figures

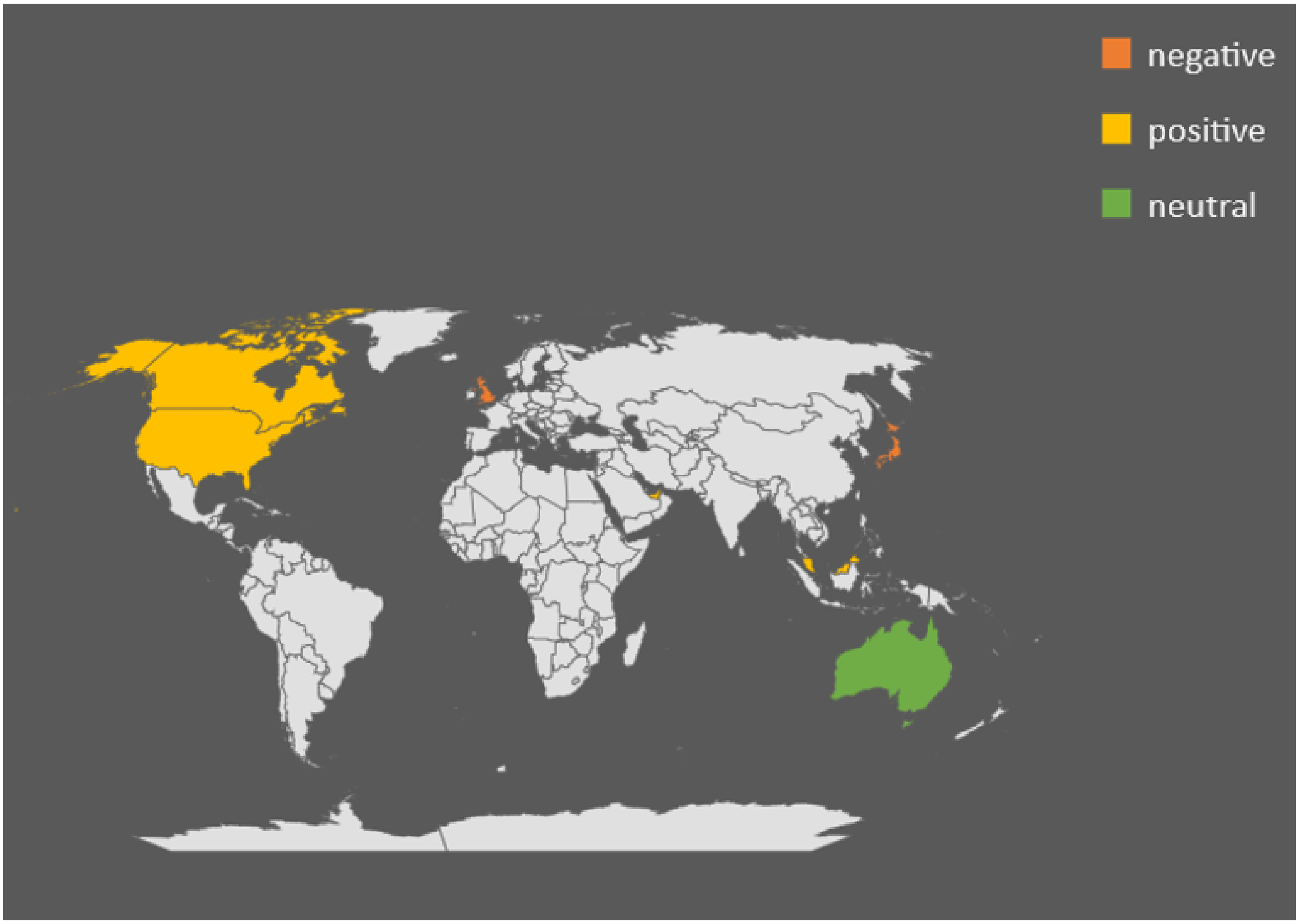

Figure 1

Distribution of tweet sentiments geographical locations

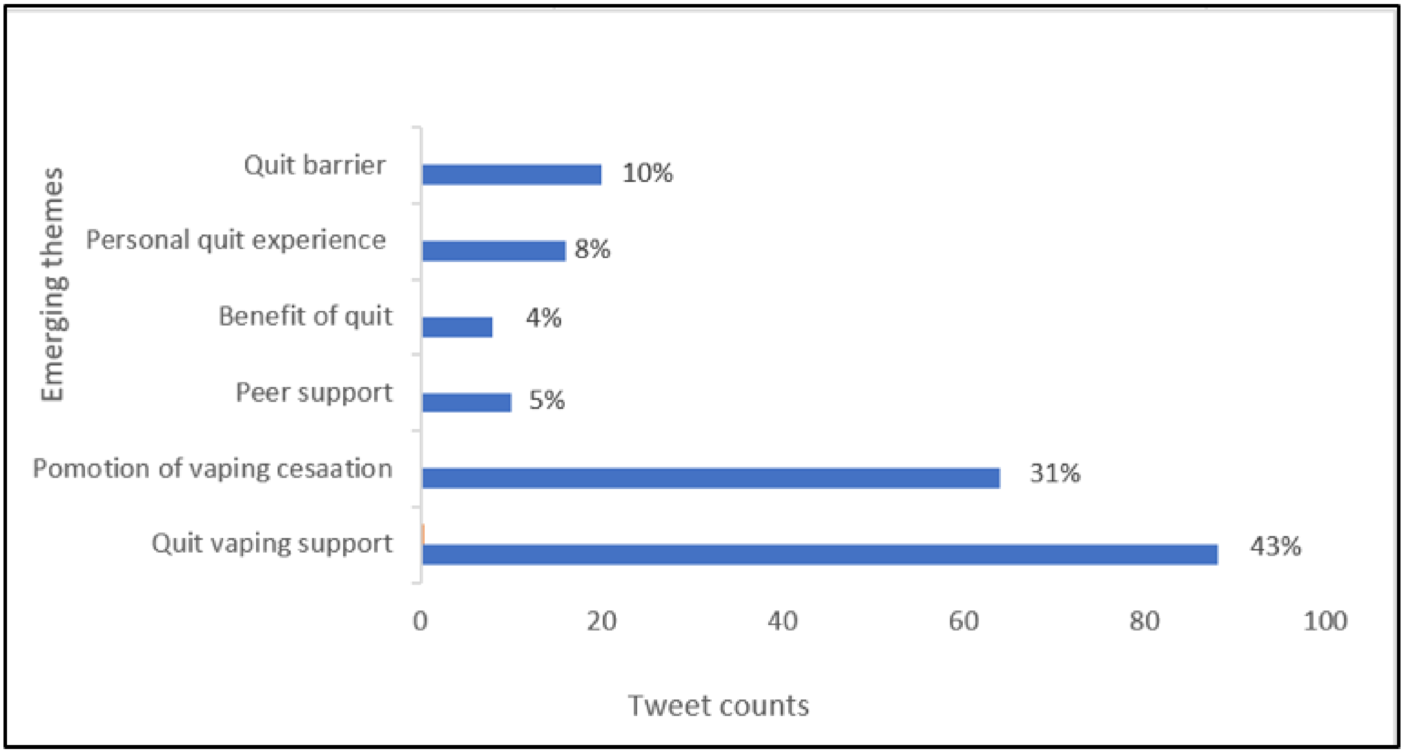

Figure 2

The proportion of tweets and emergent themes
